# Supplementary material for: Predicting visual function by interpreting a neuronal wiring diagram
Source: Nature. 2024 Oct 2;634(8032):113–23. doi: 10.1038/s41586-024-07953-5 (PMC11446822; doi:10.1038/s41586-024-07953-5)
Supplement: Supplementary file 6 — CRF and ERF predictions for individual TmY4 and TmY9 cells. Analogous to Supplementary Data 3, but for TmY target types. Shown are the top four monosynaptic pathways, the strongest pathway passing through each of the top ten intermediary types (ranking from Extended Data Fig. 7), and the trisynaptic pathway Tm1–TmY–Dm3–TmY (see the section entitled Prediction of spatial normalization). [file 41586_2024_7953_MOESM6_ESM.zip › DataS4/TmY9qΓèÑ/720575940632349838.pdf]

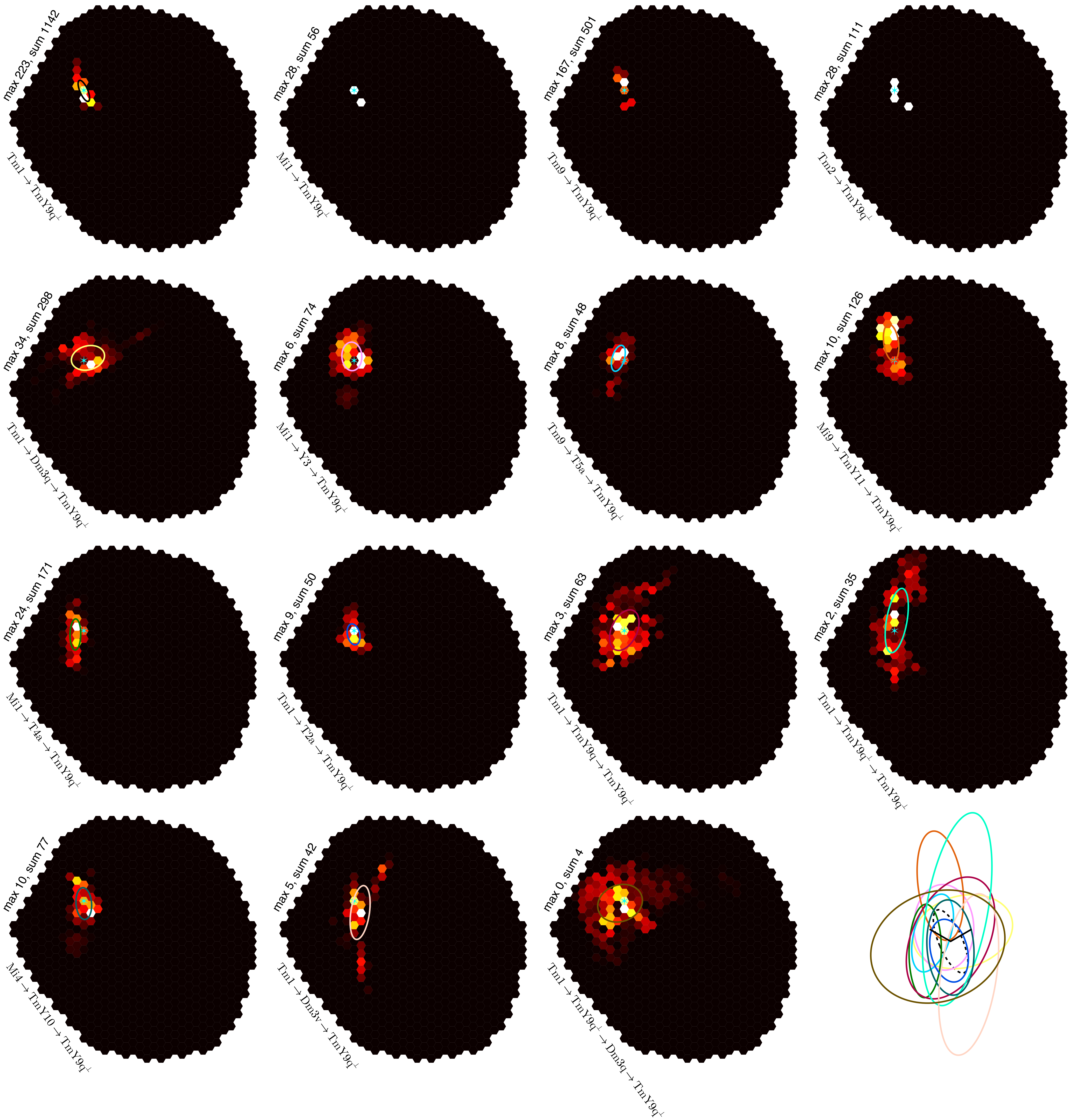

max 223, sum 1142
Tm1 → TmY9q⊥
max 28, sum 56
Mi1 → TmY9q⊥
max 167, sum 501
Tm9 → TmY9q⊥
max 28, sum 111
Tm2 → TmY9q⊥
max 34, sum 298
Tm1 → Dm3q → TmY9q⊥
max 6, sum 74
Mi1 → Y3 → TmY9q⊥
max 8, sum 48
Tm9 → T5a → TmY9q⊥
max 10, sum 126
Mi9 → TmY11 → TmY9q⊥
max 24, sum 171
Mi1 → T4a → TmY9q⊥
max 9, sum 50
Tm1 → T2a → TmY9q⊥
max 3, sum 63
Tm1 → TmY9q → TmY9q⊥
max 2, sum 35
Tm1 → TmY9q⊥ → TmY9q⊥
max 10, sum 77
Mi4 → TmY10 → TmY9q⊥
max 5, sum 42
Tm1 → Dm3v → TmY9q⊥
max 0, sum 4
Tm1 → TmY9q⊥ → Dm3q → TmY9q⊥
